# The systematic role of pancreatic cancer exosomes: distant communication, liquid biopsy and future therapy

**DOI:** 10.1186/s12935-024-03456-5

**Published:** 2024-07-25

**Authors:** Cheng Qin, Tianyu Li, Chen Lin, Bangbo Zhao, Zeru Li, Yutong Zhao, Weibin Wang

**Affiliations:** Department of General Surgery, State Key Laboratory of Complex Severe and Rare Diseases, Peking Union Medical College Hospital, Chinese Academy of Medical Sciences and Peking Union Medical College, Beijing, China

**Keywords:** Pancreatic cancer, Extracellular vesicles, Exosomes, Tumor microenvironment, Liquid biopsy

## Abstract

Pancreatic cancer remains one of the most lethal diseases worldwide. Cancer-derived exosomes, benefiting from the protective role of the lipid membrane, exhibit remarkable stability in the circulatory system. These exosomes, released by tumor microenvironment, contain various biomolecules such as proteins, RNAs, and lipids that plays a pivotal role in mediating distant communication between the local pancreatic tumor and other organs or tissues. They facilitate the transfer of oncogenic factors to distant sites, contributing to the compromised body immune system, distant metastasis, diabetes, cachexia, and promoting a microenvironment conducive to tumor growth and metastasis in pancreatic cancer patients. Beyond their intrinsic roles, circulating exosomes in peripheral blood can be detected to facilitate accurate liquid biopsy. This approach offers a novel and promising method for the diagnosis and management of pancreatic cancer. Consequently, circulating exosomes are not only crucial mediators of systemic cell-cell communication during pancreatic cancer progression but also hold great potential as precise tools for pancreatic cancer management and treatment. Exosome-based liquid biopsy and therapy represent promising advancements in the diagnosis and treatment of pancreatic cancer. Exosomes can serve as drug delivery vehicles, enhancing the targeting and efficacy of anticancer treatments, modulating the immune system, and facilitating gene editing to suppress tumor growth. Ongoing research focuses on biomarker identification, drug delivery systems, and clinical trials to validate the safety and efficacy of exosome-based therapies, offering new possibilities for early diagnosis and precision treatment in pancreatic cancer. Leveraging the therapeutic potential of exosomes, including their ability to deliver targeted drugs and modulate immune responses, opens new avenues for innovative treatment strategies.

## Background

Despite amounts of effort thrown into medical research, pancreatic ductal adenocarcinoma, generally named pancreatic cancer, is still one of the most severe malignant diseases. According to the latest GLOBOCAN report, pancreatic cancer has one of the poorest prognoses among cancers, ranking as the sixth leading cause of cancer mortality. There were an estimated 510,566 new cases and 467,005 deaths attributed to pancreatic cancer worldwide [1].

Compared with the magnificent progress in the treatments against other cancer types, the 5-year survival rate of pancreatic cancer remains about 10% [2]. Until today, radical operation provided the only way to cure pancreatic cancer. However, due to the hidden symptom in the early stage, over 80% of patients lose the opportunity to receive an operation because of distant metastasis or obvious vessel invasion [3, 4]. Therefore, systematic therapy combining surgery and chemotherapy is still the main manner to manage pancreatic cancer.

The first-line chemotherapy regimens for pancreatic cancer vary based on the treatment stage and patient condition. Neoadjuvant chemotherapy for locally advanced pancreatic cancer typically includes FOLFIRINOX or gemcitabine plus nab-paclitaxel, aiming to shrink tumors and facilitate surgical resection [5–7]. Adjuvant chemotherapy post-surgery often involves gemcitabine alone or combined with capecitabine, or mFOLFIRINOX for physically fit patients, to reduce recurrence risk [8]. For metastatic pancreatic cancer, palliative chemotherapy options include FOLFIRINOX or gemcitabine plus nab-paclitaxel for those in good health, and gemcitabine monotherapy for frail patients, aiming to alleviate symptoms, extend survival, and improve quality of life [9, 10]. However, they still failed to dramatically improve the prognosis of pancreatic cancer patients due to chemoresistance [11]. In addition to chemotherapy, radiotherapy, another approved method to treat a variety of solid tumors, currently serves as a palliative manner to relieve cancerous pain, but with undesirable side effects [12].

In addition to conventional chemotherapy, several novel treatment modalities are being explored for pancreatic cancer, including immunotherapy, targeted therapy, and other systemic treatments [13]. Immunotherapy has emerged as a promising approach in oncology, but its success in pancreatic cancer has been limited. Targeting focal adhesion kinase (FAK) with defactinib, combined with pembrolizumab and gemcitabine, was investigated in a phase I study for PDAC [14]. They reported an 80% disease control rate in refractory PDAC patients, yielding a median PFS of 3.6 months and OS of 7.8 months. In the maintenance cohort, the disease control rate was 70%, with a median PFS of 5.0 months and OS of 8.3 months. Another phase 1b/2 study tested the safety and antitumor activity of niraparib combined with either nivolumab or ipilimumab in advanced pancreatic cancer. The niraparib plus ipilimumab group achieved a 6-month progression-free survival (PFS) of 59.6%, while the niraparib plus nivolumab group showed a PFS of 20.6% [15]. Targeted therapies aim to exploit specific genetic mutations or pathways involved in cancer growth. The PARP inhibitor olaparib has shown promise in patients with BRCA-mutated pancreatic cancer. In the POLO trial, olaparib as maintenance therapy for germline BRCA-mutated metastatic pancreatic cancer led to a significant improvement in PFS (7.4 months with olaparib vs. 3.8 months with placebo), though the OS benefit was not statistically significant [16]. Other systemic treatments include approaches such as oncolytic virus therapy and cancer vaccines. Oncolytic viruses are designed to selectively infect and kill cancer cells, and preliminary trials have shown some promise in pancreatic cancer [17, 18]. In the phase 1 AMPLIFY-201 study, cancer vaccine ELI-002 2P showed no dose-limiting toxicities and induced mKRAS-specific T cell responses in 84% of patients, with a median relapse-free survival (RFS) of 16.33 months. The study found a significant correlation between T cell responses and tumor biomarker reduction, indicating that ELI-002 2P is safe and effective in inducing T cell responses in KRAS-mutated tumors [19].

Despite the array of novel therapeutic approaches being investigated, the prognosis for pancreatic cancer patients remains poor, with worsening social and physical quality of life [13, 20]. Therefore, effective therapy against pancreatic cancer is urgently needed to solve the current dilemma.

As the prerequisite, understanding pancreatic cancer clearly and thoroughly is the basis for developing novel therapy. Compared to massive previous studies on malignant cells and tumor microenvironment [21], local pancreatic tumor also distantly communicates with other tissue and organs, affecting the tumor-host macroenvironment [22]. Therefore, the communication among them is vital for pancreatic cancer progression [23].

**Fig. 1:**
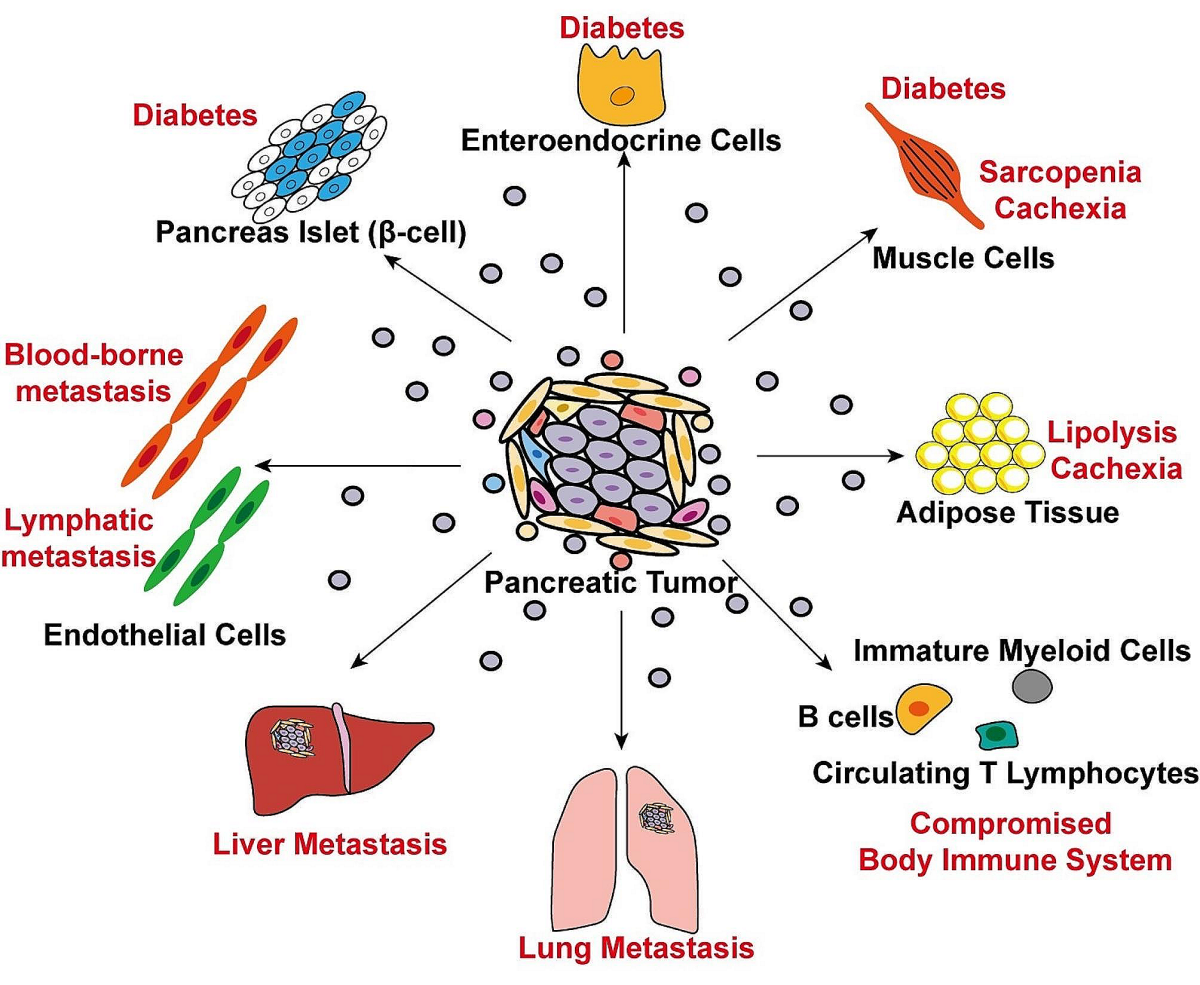
The communication between pancreatic tumor and other organs or tissues via exosomes. Proteins and RNAs in exosomes from local pancreatic tumor could be transmitted to distant organs and tissues, promoting distant metastasis, diabetes, cachexia, and compromised immune system

Extracellular vesicles, including exosomes, microvesicles, and apoptotic bodies, differ significantly in their physical characteristics, biological functions, and origins [24–26] and are extensively studied for their potential diagnostic applications in pancreatic cancer [27]. Exosomes (30–150 nm) are small, spherical vesicles formed via the endosomal pathway, playing roles in intercellular communication and genetic information transfer. Microvesicles (100-1,000 nm) bud directly from the plasma membrane and are involved in cell signaling and inflammation. Apoptotic bodies (50 − 5,000 nm) arise from cells undergoing programmed cell death and contain cellular debris, aiding in the clearance of apoptotic cells. Compared to other extracellular vesicles, exosomes are the most extensively studied. They can be released by all cell types and are found in various body fluids, including serum, blood plasma, and urine. Exosomes contain proteins, metabolites, and nucleic acids, making them a vital mediator in regulating distant cell-cell communication [28] (Fig. 1).

Benefiting from the Protective lipid bilayer membrane with identifiable proteins (such as CD9, CD63, CD81), exosomes could mediate adjacent and long-distance cell-cell communication in both health and disease compared to other secretory molecules [29]. Originally, exosomes are derived from endosomes. After encapsulating biomolecules from cytosol through inward budding, late endosome turns to be multivesicular body (MVB) containing massive intraluminal vesicles. Then, some intracellular MVB degrade to lysosomes, while others fuse with the cell membrane and release contained exosomes to extracellular space [30]. Such biosynthesis of exosomes in cancer cells is finely regulated by many molecules under different conditions [31]. Although the precise physiology of exosomes biogenesis remains not very clear, their role in facilitating tumorigenesis is relatively widely researched.

Circulating exosomes have significant utility in cancer diagnostics [32]. They carry tumor-specific proteins, RNA, and DNA, enabling early detection and non-invasive diagnosis. As a noninvasive detection method, liquid biopsy offers notable benefits over traditional diagnostic procedures such as tissue biopsy, including enhanced cost-efficiency and convenience [33]. Exosomes can be stably present in bodily fluids, including blood, urine, and saliva, making them highly promising for liquid biopsy [34]. Exosomes can also deliver therapeutic molecules directly to cancer cells, enhancing treatment efficacy and reducing side effects. Additionally, analyzing exosomal content provides insights into tumor biology and drug resistance mechanisms, aiding in the development of personalized treatment strategies and real-time monitoring of therapeutic responses [35]. Moreover, artificially modified exosomes could also be employed as nanocarriers in anticancer therapy [36]. Given the complex tumor microenvironment and poor prognosis of pancreatic cancer, research on exosome-based liquid biopsies may offer more effective diagnostic and prognostic methods for this disease [37]. Exploring the application of exosomes in pancreatic cancer not only helps improve early detection rates but also provides new approaches for personalized treatment and improving survival rates.

In this review, we summarized current knowledge about exosomes in mediating distant cell-cell communication in pancreatic cancer. In addition, the potential clinical application and future research directions of exosomes in pancreatic cancer was also introduced.

## Exosomes connecting local tumor microenvironment with other organs/tissues

### Body immune system

**Fig. 2:**
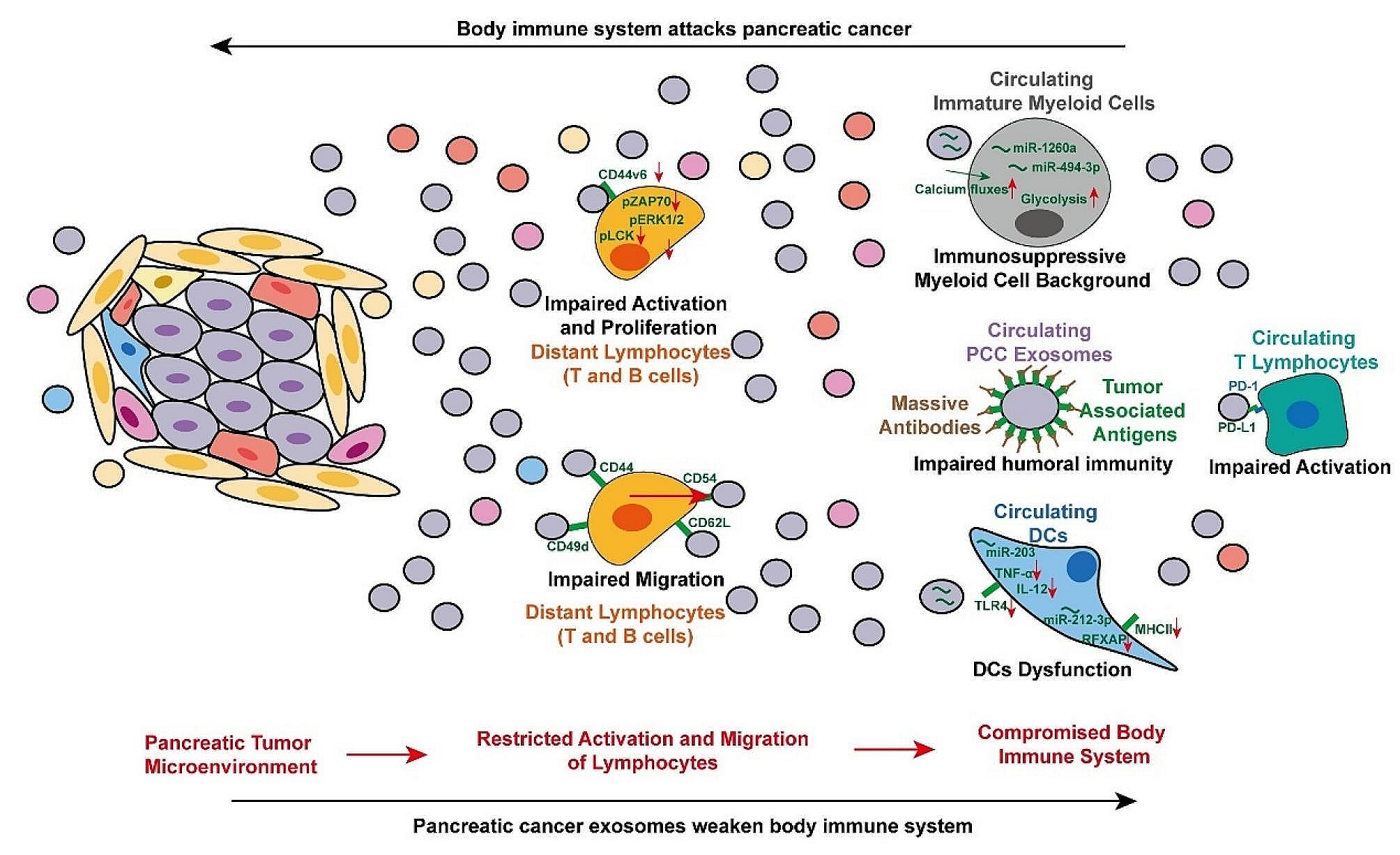
Impact of Pancreatic Cancer Exosomes on the Immune System and Tumor Microenvironment. This illustration depicts the complex interplay between pancreatic cancer cells and the body’s immune system, highlighting how pancreatic cancer exosomes weaken immune responses. On the left, the pancreatic tumor microenvironment restricts the activation and migration of lymphocytes, leading to impaired activation and proliferation of distant T and B lymphocytes (center). The diagram also shows circulating immature myeloid cells creating an immunosuppressive background, and pancreatic cancer cell (PCC) exosomes impairing humoral immunity through massive antibodies and tumor-associated antigens. On the right, circulating dendritic cells (DCs) exhibit dysfunction and humoral immune is impaired, contributing to a compromised immune system. Additionally, circulating T lymphocytes show impaired activation, exemplified by PD-1 and PD-L1 interactions. The overall effect is a compromised body immune system that struggles to effectively attack pancreatic cancer

Immune cells engage in comprehensive and dynamic crosstalk with tumor cells in the pancreatic TME via exosomes [38, 39]. For instance, LncRNA SBF2-AS1 in M2 macrophages-derived exosomes endogenously compete and suppress miR-122-5p expression in PCCs upon uptake, thus promoting XIAP protein level and accelerating PC development [40]. Similarly, M2 macrophages release miR-193b-3p [41], miR-202-5p and miR-142-5p [42] to promote the proliferation, invasion, and migration of PC. Meanwhile, PC cell-derived exosome miR-210 can promote macrophage M2 polarization via targeting and inhibiting FGFRL1, thereby activating the p-PI3K/p-AKT/p-mTOR pathway and promoting chemoresistance [43]. PC cells also generate Exosome lncRNA, such as FGD5-AS1, which induces M2 macrophage polarization and promote PC cells’ malignant behaviors [44]. Thanks to the protective effect of the lipid bilayer, exosomes secreted by PC can also act on immune cells in the circulatory system as well as in other organs/tissues (Fig. 2).

A previous study on rats showed that murine PCCs-derived exosomes could be internalized of a variety of leukocytes located in peritoneal exudate, spleen, peripheral blood, bone marrow, and lymph nodes. Upon uptake, exosomes can hinder leukocytes activation and proliferation via down-regulating of CD44v6 and inhibiting phosphorylation of LCK, ZAP70 and ERK1/2 [45]. Also, they can occupy migration-associated ligands, such a as CD44, CD49d, CD54, and CD62L [46, 47], and significantly impair lymphocyte migration. Besides, the functions of CTLs, NKs, and tumor-lysate-loaded DCs were activated, supporting immune response induction of pancreatic cancer [45]. However, in circulating system, exosomes secreted by PCCs, especially those without SMAD4 expression, create an immunosuppressive microenvironment characterized by unbalanced immature myeloid cells by upregulating calcium fluxes and glycolysis, which potentially mediated by exo-miRNAs miR-1260a and miR-494-3p [48].

Additionally, PCCs-derived exosomes were taken up by circulating T lymphocytes, activating p38 mitogen-activated protein kinase and inducing endoplasmic reticulum stress-mediated apoptosis of recipient cells, which finally caused immunosuppression [49]. For instance, exosomal PD-L1 could inhibit circulating cytotoxic T lymphocytes and promote PC invasion [50, 51]. In addition to the cytotoxic immune response, the humoral immune response has a more large-scale battlefield involving immune tissue/organs, circulating system, and local tumor. PCCs-derived exosomes preferred to equip enriched tumor associated antigens (TAAs) on their surface and induce B cells-mediated humoral immune response [52]. However, those exosomes could absorb massive autoantibodies as a decoy and facilitate PCCs to escape from complement-mediated cytotoxicity [53].

Furthermore, PCCs-derived exosomes have been demonstrated to inhibit the immune system by deregulating DCs in PBMC. After being taken up by DCs, miR-203-containing exosomes generated by PC cells can boost intracellular miR-203 levels and suppress the production of TLR4 and downstream cytokines (TNF-α and IL-12), finally causing their dysfunction [54]. Zech et al. revealed that PCCs-derived exosomes acquired by DCs can effectively suppress IL-2/PI3K/Akt signal pathway in the lymphocytes, which activated apoptosis [45]. Also, PCCs-derived exosomes suppress RFXAP expression via miR-212-3p, resulting in decreased MHC II expression and immunological tolerance of DCs [55].

The resistance that pancreatic cancer develops to immunotherapy is partly due to the accumulation of immunosuppressive cells in the TME. Wong et al. recently discovered that myeloid-derived suppressor cells (MDSC) formation in PC could be induced by macrophage migration inhibitory factor (MIF) from the tumor-derived exosome. They also synthesized a MIF tautomerase inhibitor, which effectively inhibited exosome-induced MDSC differentiation and tumor growth in an orthotopic PC model by promoting CD8 + T cells infiltration in the TME [56].

### Distant metastasis

The movement of tumor cells from vessel to tissue is important, thus the first step of distant metastasis. PCCs could transmit tissue factors to endothelial cells through circulating exosomes. With the help of factor VIIa and factor Xa, those exosomes upregulated endothelial E-selectin and IL-8 synthesis, inducing pro-adhesive and pro-inflammatory phenotype of endothelial cells and promoting metastasis [57]. Additionally, exosomes circular RNA IARS (circ-IARS) from PCCs significantly enhanced endothelial monolayer permeability and promoted blood-borne distant metastasis as well [58]. Moreover, another recent study showed that hypoxic PCC secreted exosomal miR-30b-5p targeted GJA1 in endothelial cells, promoting angiogenesis and distant metastasis [59]. However, PCC also could transmit exosomal miR-29b to endothelial cells and repressed ROBO1 and SRGAP2, which inhibited angiogenesis and metastasis [60].

Lymphatic system is an important defense system of the human body, consisting of lymphatic vessels, lymphoid tissue, and lymphoid organs [61]. Current research is being devoted to understand how PC cells transfer to the lymph nodes. A potential mediator that causes lymphatic metastasis is exosomes [62–64]. Exosomal lnRNA LNMAT2 was proved to facilitate lymph-angiogenesis and lymphatic metastasis in bladder cancer via VEGF-C [65]. Also, cancer-secreted exosomal miRNAs could promote lymphatic metastasis in esophageal [66, 67], cervical [68], endometrial [69], gastric [70], and breast cancer [71]. In pancreatic cancer, Zhou et al. found that PCC-derived exosomes could promote lymph-angiogenesis via downregulating ABHD11-AS1 expression and upregulating proliferation, migration, and tubes formation in lymphatic endothelial cells [72].

**Fig. 3:**
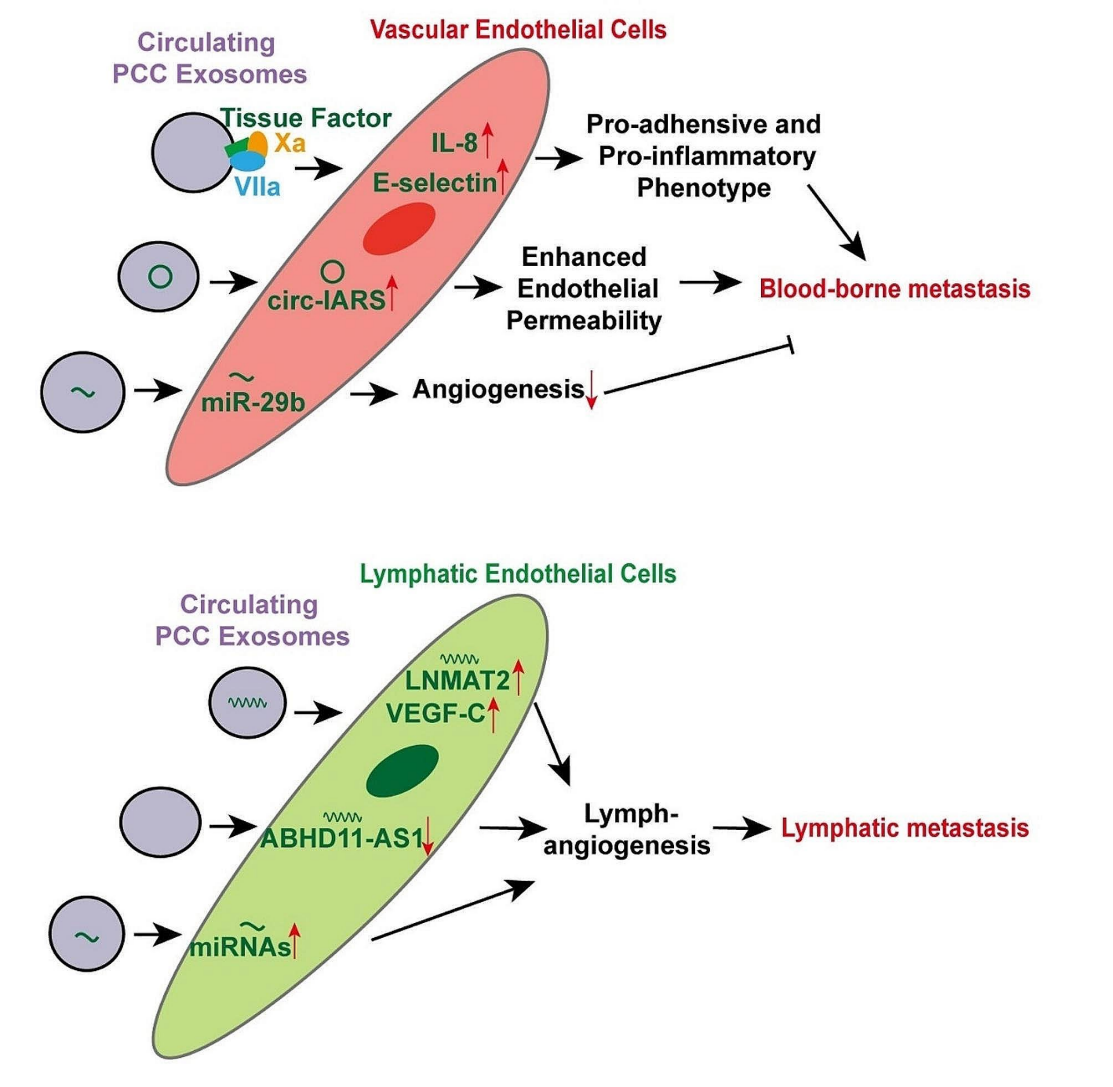
Mechanisms by which circulating PCC-derived exosomes act on vascular and lymphatic endothelial cells to promote metastasis

**Fig. 4:**
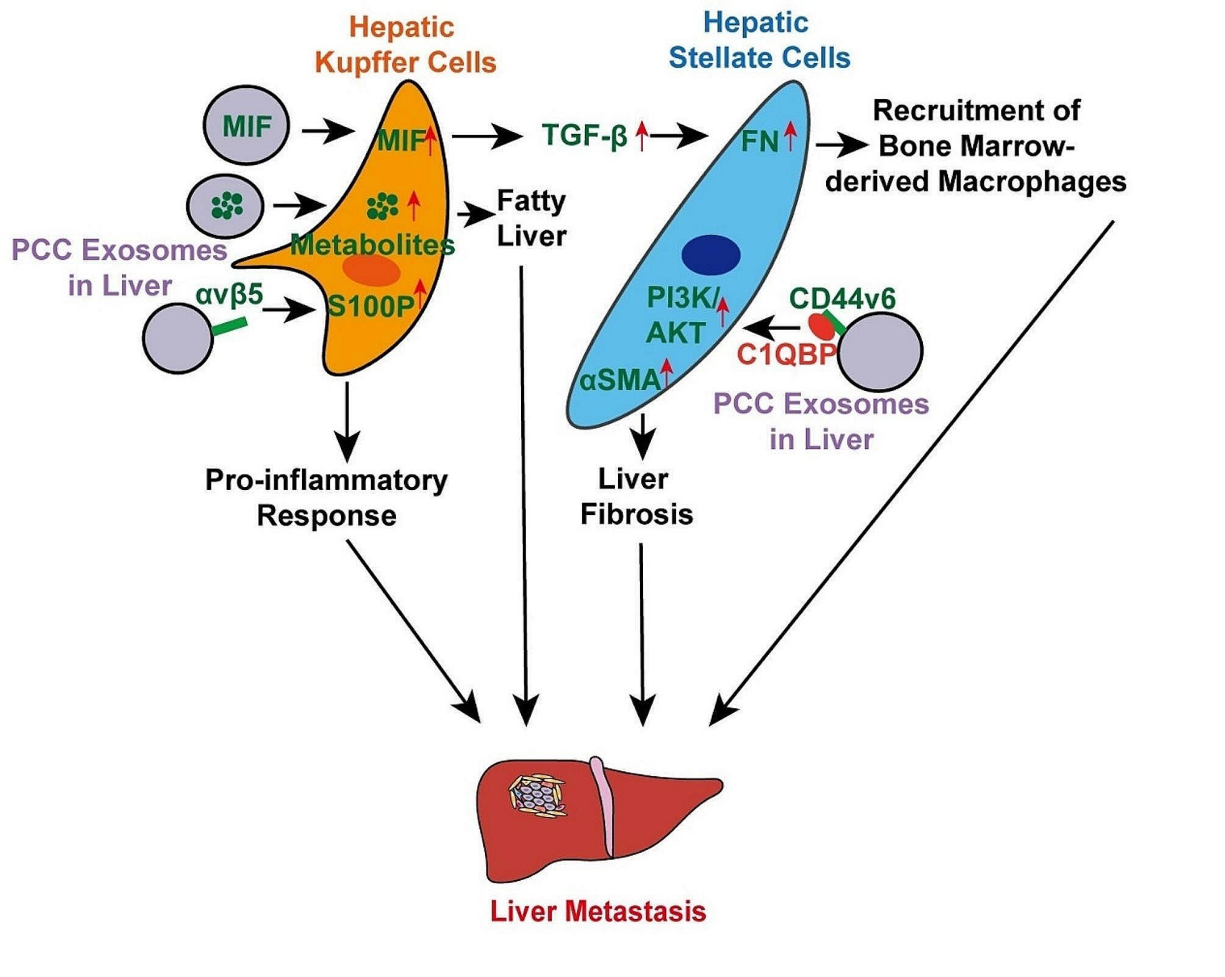
Mechanisms by which circulating PCC-derived exosomes act on a hepatic Kupffer cells and stellate cells to promote liver metastasis

Besides the impact on vascular endothelial cells and lymphatic system (Fig. 3), the direct effects of exosomes on target organs were also significant to construct premetastatic niche. The disposition of PCC-derived exosomes in target organs, such as macrophages in lung and bone marrow, could be detected before any metastases are present [73]. The underlying molecular mechanism of exosomes in driving distant metastasis is being gradually revealed. Liver, peritoneum, and lung are common metastatic sites in pancreatic cancer patients [74]. The pattern of PCC-derived exosomes involvement in liver metastasis is illustrated in Fig. 4. Primary tumor cells could secrete exosomes to modify the extracellular matrix of other organs, rendering them to be receptive to metastatic seeding. For example, specific integrins on PCC-derived exosomes could drive different metastasis. Exosomes with integrin αvβ5 mediated liver tropism whereas α6β4 and α6β1 contributed lung tropism, which could induce pro-inflammatory pathways in recipient cells and create pre-metastatic niches in target organs [75]. Macrophage migration inhibitory factor (MIF), enriched in pancreatic tumor exosomes, could impel hepatic Kupffer cells to secrete and release TGF-β. Then, the fibronectin production of hepatic stellate cells was upregulated, which increased the recruitment of bone marrow-derived macrophages and promoted liver pre-metastatic niche formation [76]. Besides the stimulation mediated by Kupffer cells, PCC-derived exosomes containing CD44v6 and complement C1q binding protein (C1QBP) complex contributed to the phosphorylation of PI3K/AKT pathway and α-SMA expression in hepatic stellate cells directly, which enhanced liver fibrosis and liver metastasis of pancreatic cancer [77]. Additionally, another recent report suggested that PCCs-derived exosomes could reprogram the transcriptome of CD11b + cells in bone marrow, which might subsequently turn to be bone marrow-derived macrophages within tumor microenvironment and liver premetastatic niches [78]. Also, the PCCs-derived exosome tRF-GluCTC-0005 was able to recruit MDSCs and activated hepatic stellate cells in the liver, which created an immunosuppressive microenvironment and further promoted liver metastasis [79]. Moreover, PCC also transferred exosomes with metabolites, particularly palmitic acid, to Kupffer cells in liver, inducing fatty liver and systemically dysregulated metabolism in pancreatic cancer patients. As a result, the systemic immune functions were compromised, and the distant metastasis was accelerated [80].

Except for promoting liver metastasis, PCCs with low protein kinase D1 (PKD1) expression preferred to secret α6β4 positive exosomes, which promoted lung metastasis of pancreatic cancer [81]. Additionally, PCCs with mutant p53 released exosomes containing certain podocalyxin levels, which could influence the motility of fibroblasts and promote the formation of pro-invasive extracellular matrix in distant organs, such as lung [82]. After injection of PC ascites-derived exosomes from PC patients in mice, the vascular permeability in the lung was promoted, leading to the extravasation and colonization of PCCs [83]. Compared with liver and lung metastasis, the underlying mechanism of possible exosome-mediated peritoneal metastasis in pancreatic cancer remains to be elucidated.

### Diabetes

**Fig. 5:**
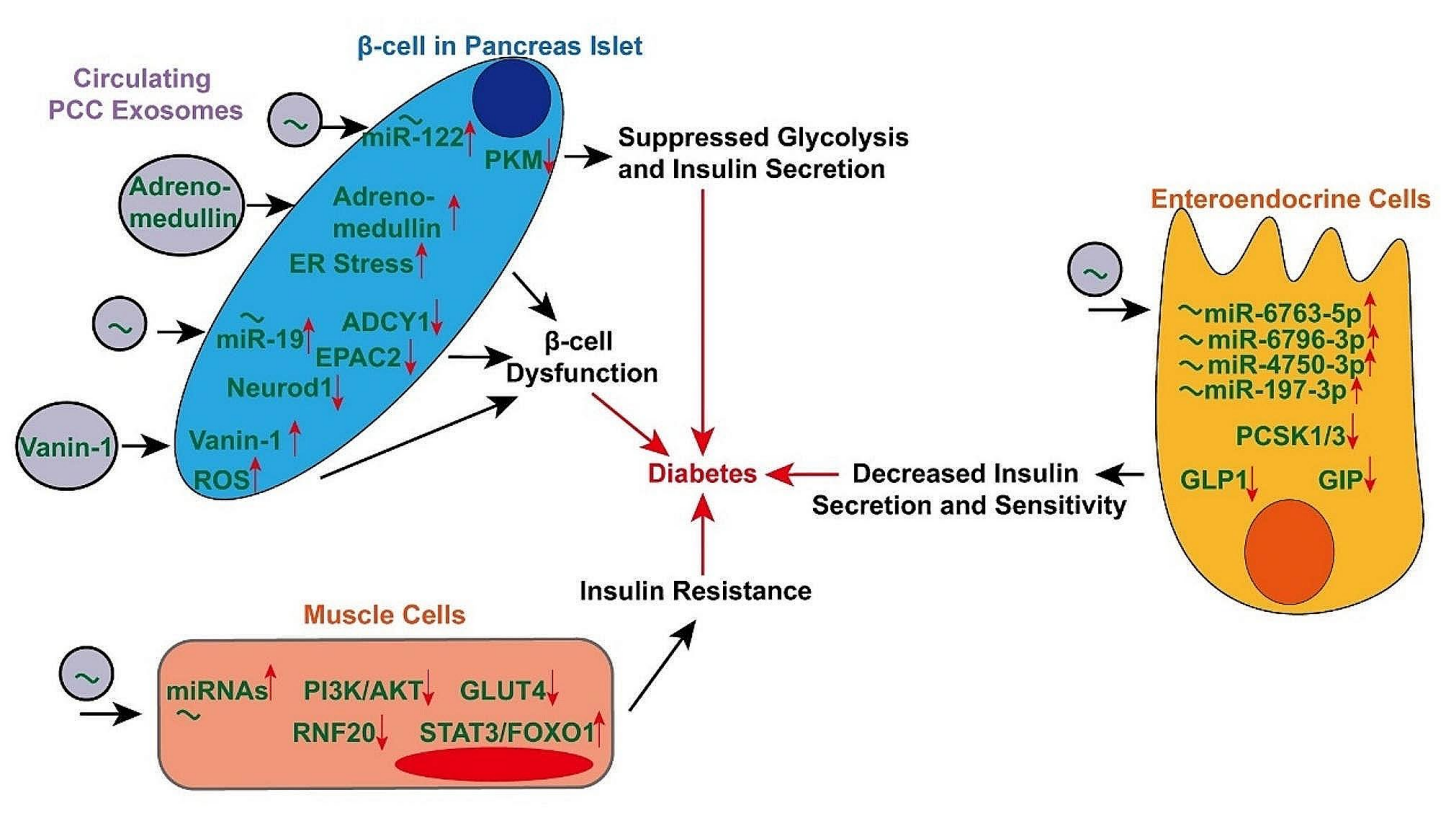
Mechanisms by which circulating PCC-derived exosomes act on pancreatic β-cells, muscle cells and enteroendocrine cells to promote the development of diabetes

Extensive epidemiological evidence suggested that diabetes could serve as both a risk factor and clinical manifestation of pancreatic cancer [84, 85]. However, the underlying molecular mechanism between pancreatic cancer and diabetes remains unclear [86]. PCC-derived exosomes may cause changes in the whole pancreatic islets including intra-tumoral islets, resulting in the occurrence of pancreatic cancer-related diabetes [87, 88]. In addition, the development of diabetes is not only related to the pancreatic islets, but also to the sugar uptake and fat metabolism of peripheral tissues such as muscle tissue [89]. We have revealed the biological mechanisms of exosomes in mediating this process (Fig. 5).

Tumor-derived EVs could transfer miR-122 to β-cells and target pyruvate kinase M (PKM), thus suppressing glycolysis and insulin secretion, promoting glucose tolerance, fasting hyperglycemia, eventually contributing to the impairment of whole-body glucose homeostasis [90]. In PC, adrenomedullin was up-regulated and responsible for β-cells insulin resistance [91]. PCCs-derived exosomes transmitted adrenomedullin to β-cells, which caused endoplasmic reticulum stress and β-cell dysfunction. Therefore, insulin secretion was indirectly suppressed by PCCs [92]. Also, adrenomedullin can be loaded into exosomes derived from cancer-associated fibroblasts, and then delivered to recipient adipocytes to enhance lipolysis in the adipose tissues [93], which was reckoned as a hallmark of diabetes [94]. MiR-19a contained in PCCs-derived exosomes was also reported to regulate insulin secretion. It was found that miR-19a induced β-cell dysfunction via targeting ADCY1 and EPAC2 [95], as well as inhibited insulin production via targeting Neurod1 in pancreatic β cells [96]. Additionally, another recent report showed that Vanin-1 in PCC-derived exosomes could impair adjacent islets by producing oxidative stress and β-cell dedifferentiation, leading to diabetes [97]. In addition, the secretion and function of insulin were affected by many other regulators. Glucagon-like peptide-1 (GLP-1) and glucose-dependent insulinotropic peptide (GIP) are two important hormones contributing to glucose homeostasis through enhancing insulin secretion and sensitivity. Exosomes miRNAs (miR-6796-3p, miR-6763-5p, miR-4750-3p, and miR-197-3p) from PCCs could suppress proprotein convertase subtilisin kexin type 1/3 (PCSK1/3) in enteroendocrine cells, subsequently inhibiting GLP1 and GIP secretion [98].

Decreased peripheral glucose use also contributes to the pathogenesis of diabetes. PCCs-derived exosomes could inhibit the PI3K/AKT signaling pathway in muscle cells, resulting in compromised expression of glucose transporter 4 protein (GLUT4) and insulin resistance [99]. Besides, Wang et al. proved that PCCs-derived exosomal miR-let-7b-5p stimulated insulin resistance in skeletal muscle cells via targeting RNF20 and activating STAT3/FOXO1 pathway [100].

### Cachexia

Cachexia is a prominent and frequent clinical feature of pancreatic cancer, which is mainly characterized by non-intentional weight loss, such as sarcopenia and adipopenia [101]. Importantly, cachexia is associated with poor life quality and even death in pancreatic cancer patients, attracting massive attention from clinicians and nutritionists [101]. As another systematic syndrome besides diabetes, the cachexia process in pancreatic cancer could also be partially attributed to exosomes.

Weight loss and malnutrition due to cachexia are often caused by muscle, fat, and even bone catabolism. Tumor-released extracellular surface heat shock protein 70 (HSP70) and Hsp90 were identified as key factors causing myotube atrophy and muscle wasting [102–104]. PCCs released markedly increased levels Hsp70/Hsp90-containing EVs, which targeted in myotubes and resulted in the loss of the myosin heavy chain and myotube diameter via upregulating of p38 MAPK, C/EBPβ, and LC3-II [103]. Another study by Yang et al. reported that ZIP4 could induce muscle loss and cachexia by activating CREB-regulated expression of RAB27B, a key regulator required for EVs releasing in PCCs. Those exosomes containing high levels of HSP70 and HSP90 could stimulate Toll-like receptor 4 (TLR4) on the recipient muscle cells, finally promoting p38 MAPK-mediated muscle catabolism and cachexia [105].

Additionally, the abovementioned adrenomedullin in PCCs-derived exosomes could activate ERK1/2 and p38 MAPK pathways in adipocytes, contributing to lipolysis in adipose tissue and weight loss [106]. Another study by Shibata et al. reported that intravenous injection of PCCs-derived EVs could promote lipolysis and negatively affect body weight in mice model. They also revealed that EVs in PC patients’ serum exhibit higher ITGA6 and ITGB1 expression levels, which allowed them to be preferentially distributed to the adipose tissue [107].Moreover, compared with the healthy control, the expression of genes for browning, lipolysis, fibrosis, and acute inflammation was upregulated in subcutaneous adipocytes exposed to plasma exosomes from pancreatic cancer patients [108].

A recent report also suggested that PCCs-derived exosomal miR-125a-5p could induce osteoclast differentiation and cancer-related bone loss [109], further indicating the role of exosomes in pancreatic cancer patients accompanied with cachexia.

## Clinical values of exosomes in diagnosis, monitoring, and therapy

### Diagnosis and monitoring

Due to non-specific symptoms at early stage, about 80% of pancreatic cancer patients are diagnosed to be the advanced stage when initially evaluated. Therefore, early detection is significant and promising for improving the dismal prognosis [110, 111]. Benefiting from the protective role of lipid bilayer membrane and CD47 transmembrane protein, exosomes carrying specific information about pancreatic cancer is relatively stable in the circulating system and less likely eliminated by monocytes [112], providing the possibility of early diagnosis via liquid biopsy (Table 1).

**Table 1:** Diagnosis and monitoring the status of pancreatic cancer patients via Exosomes

Countries	Samples	Exosomes isolation	Exosomes markers	Applications	References
Germany	Serum	Ultracentrifugation	GPC1	Diagnosis	[115]
China	Plasma	Density gradient ultracentrifugation	GPC1, CD82	Diagnosis	[117]
USA	Plasma	Ultracentrifugation	miR-10b, miR-21, miR-30c, miR-106b, miR-20a, miR-181a, miR-483, miR-let7a, miR-122	Diagnosis	[119]
USA	Plasma	Ultracentrifugation	EGFR, EpCAM, WNT2, GPC1	Diagnosis	[118]
China	Serum	Polymer-based precipitation	ZIP4	Diagnosis	[124]
USA	Plasma	Magnetic bead-based isolation	mRNAs (CK18, CD63), miR-409	Diagnosis	[121]
China	Plasma	Ultracentrifugation	long RNA (FGA, KRT 19, HIST1H2BK, ITIH2, MARCH2, CLDN1, MAL2 and TIMP1)	Diagnosis	[122]
USA	Serum	Polymer-based precipitation	miR-191, miR-21, miR-451a	Diagnosis	[123]
China	Serum	Membrane-based affinity	miR-451a	Diagnosis	[128]
China	Plasm	Membrane-based affinity	miR-19b	Diagnosis	[129]
Japan	Serum	Not Given	mRNAs (WASF2, ARF6) snoRNAs (SNORA74A, SNORA25)	Diagnosis	[126]
USA	Saliva, Serum	Magnetic bead-based isolation	mRNAs (Apbblip, Aspn, Incenp, Daf2, Foxp1)	Diagnosis	[136]
USA	Plasma, Serum	Ultracentrifugation	miR145-5p, miR200b-3p, miR429, miR1260b, miR145-3p, miR216b-5p, miR200a-3p, miR217-5p	Diagnosis	[120]
USA	Plasma	Polymer-based precipitation	EphA2	Diagnosis, Reflecting chemotherapy response	[125]
China	Plasma	Ultracentrifugation	ALIX	Diagnosis	[127]
Germany	Serum	Ultracentrifugation	ADAM8, miR-451, miR-720	Diagnosis	[131]
Japan	Saliva	Polymer-based precipitation	miR-1246, miR-4644	Diagnosis	[137]
China	Plasma	Ultracentrifugation	miR-335-5p, miR-340-5p	Diagnosis, Predicting metastasis and prognosis	[130]
Korea & Japan	Serum or Plasma	Polymer-based precipitation	miR-130b-5p, miR-133a-3p, miR-1273f, miR-195-5p, miR-432-5p, miR-1229-3p	Predicting early recurrence	[140]
USA	Plasma	Magnetic bead-based isolation	miR-1299, mRNA (GAPDH)	Predicting distant metastasis	[121]
China	Serum	Polymer-based precipitation	EphA2	Predicting distant metastasis	[141]
USA	Plasma	Ultracentrifugation	DNA (KRAS mutant allele fraction)	Reflecting chemotherapy response	[142]
USA	Plasma	Immune lipoplex nanoparticle (ILN) biochip assay	GPC1	Diagnosis and prognosis	[116]

Studies based on proteomics suggested that protein signatures of exosomes derived from patients’ plasma could reflect the pathophysiology of pancreatic cancer [113]. Also, the phenotype signatures of plasma-derived exosomes in pancreatic cancer patients were proved to have high diagnostic accuracy and strongly correlated with tumor stages [114]. Through analyzing the levels of glypican 1 (GPC1) exosomes in serum, a study involving German patients revealed that GPC1 exosomes could perfectly distinguish pancreatic cancer from healthy donors and individuals with benign pancreatic diseases. The AUC was 1.0 (95% CI: 0.988–1.0), with 100% sensitivity and 100% specificity [115]. By performing immune lipoplex nanoparticle biochip assay, Li et al. validated that circulating exosomes GPC1 could serve as a viable biomarker for PC which was validated in clinical cohorts at multiple hospitals [116]. While GPC1 alone showed high accuracy, combining Exosomes GPC1, CD82, and serum CA199 levels could also realize better diagnostic efficiency, achieving an AUC of 0.942 (95% CI: 0.882–1.000) [117]. Similarly, the combination of exosomal markers EGFR, EpCAM, WNT2, and GPC1 demonstrated excellent diagnostic efficiency, achieving an AUC of 1.0, with a sensitivity of 82% and a specificity of 90% [118]. However, another study suggested that circulating exosomes GPC1 was not diagnostic for pancreatic cancer, but indicated high levels of miR-181a, miR-10b, miR-21, miR-30c, and low levels of miR-let7a in plasma exosomes could effectively differentiate pancreatic cancer from healthy donors and chronic pancreatitis patients [119]. The variations between different groups’ findings can be attributed to several factors, including differences in blood preservation conditions, exosome extraction methods, protein/RNA quantification techniques, and population demographics. These factors can significantly influence the results and lead to discrepancies between studies. Currently, the role of exosomes in liquid biopsy field is gaining momentum. Nakamura et al. [120]. adopted exosome-based liquid biopsy to establish a 13 miRNAs signature which not only showed high diagnostic accuracy (AUC = 0.98 for training cohort; AUC = 0.93 for validation cohort) for all stages of PDAC but also successfully identified CA19-9 negative patients (AUC = 0.96, sensitivity = 91% and specificity = 90%), demonstrating the potential superiority than CA19-9. Besides, when combined with CA19-9 levels, the diagnostic accuracy significantly improved (AUC = 0.99 compared to AUC = 0.86 for CA19-9 alone).

Other relevant studies also suggested that plasma exosomes microRNAs (miR-409, miR-191, miR-21, miR-451a, miR-19b, miR-335-5p, miR-340-5p, miR-451,miR-720), mRNAs (CK18, CD63, FGA, KRT19, HIST1H2BK, ITIH2, MARCH2, CLDN1, MAL2, TIMP1, WASF2, and ARF6), small nucleolar RNAs (SNORA74A and SNORA25), and proteins (ZIP4, EphA2, ALIX, ADAM8) could assist to pancreatic cancer diagnosis [120–135]. Besides blood, exosomes in saliva provided information about pancreatic cancer as well. Exosomes from the saliva of mice models bearing orthotopically implanted pancreatic cancer had higher mRNAs (Apbblip, Aspn, Incenp, Daf2, and Foxp1) [136]. Furthermore, Saliva-derived exosomal miR-1246 and miR-4644 fulfilled pancreatic cancer diagnosis with 83.3% sensitivity and 92.3% specificity [137], which revealed a more convenient and non-invasive method for potentially diagnosing pancreatic cancer (Table 1).

Recently, a novel method for mutational protein analysis of single extracellular vesicle has been developed, which can capture individual extracellular vesicles and analyze multiple protein markers within them based on antibody capture and fluorescence imaging [138]. Researchers found KRAS^mut^ and P53^mut^ could serve as extracellular vesicle markers for detecting pancreatic cancer. By applying this method, PDAC with volume of merely 0.1 cubic centimeters was successfully detected, far superior to current clinical imaging detection capabilities [138].

In addition to diagnosis, accurately and dynamically monitoring the status of patients during cancer course is vital for individualized therapy and long-term survival [139] (Table 1). Lower miR-130b-5p, miR-133a-3p, miR-1273f, and higher miR-195-5p, miR-432-5p, miR-1229-3p in serum or plasma exosomes related to early recurrence after surgery in patients with pancreatic cancer [140]. Similarly, higher exosomes EphA2 in plasma also could predict early pancreatic cancer recurrence after surgery [141]. Besides predicting early recurrence, the panel containing serum exosomal miR-1299, GAPDH (mRNA), circulating mutant KRAS allele fraction, and CA199 levels could effectively predict occult distant metastasis of pancreatic cancer, which was significantly better than imaging alone [121]. Decreased exosomal EphA2 levels in plasma could reflect good/partial response to neoadjuvant therapy in pancreatic cancer patients [125]. Besides that, increased *KRAS* mutant allele fraction in exosomal DNA could predict disease progression during neoadjuvant treatment, which was superior to elevated CA19-9 levels [142].

However, there is no available assay approved by Food and Drug Administration for pancreatic cancer diagnosis and monitoring. Hence, the results of previous publications remain further validation in high-quality clinical trials. Additionally, the methods for isolating exosomes in clinical use should also be optimized and unified [143].

### Therapy

Compared with free drug and synthetic nanoparticles such as liposomes, exosomes, which are naturally generated nanoscale EVs with innate properties well suited to lipids, shuttle proteins, and nucleic acids between cells [144], can be loaded with drugs while freely traversing across dense tumor stroma, avoiding degeneration and immune clearance [145, 146]. Additionally, PCCs-derived exosomes were preferentially taken up by tumor tissue in orthotopic mice models [147, 148]. Such enhanced uptake of exosomes might be mediated by oncogenic KRAS, which promoted macropinocytosis in pancreatic cancer [112, 149]. Therefore, exosomes provided promising novel opportunities for pancreatic cancer treatment. However, their therapeutic effectiveness is restricted due to their inability to specifically target tumor cells and a high rate of clearance by the mononuclear phagocytic system. To overcome such limitations, Creeden et al. developed novel engineered “Smart Exosomes” which increased binding ability to αvβ3 on PC cells, resulting in enhanced cellular uptake and increased chemotherapy response in both in vivo and in vitro models [150].

PCCs-derived exosomes loading with gemcitabine exhibited superior results in suppressing tumor growth in xenograft mice models than free gemcitabine. Moreover, compared with free gemcitabine administrated systemically, those autologous exosomes specifically carried gemcitabine to pancreatic cancer tissue and significantly decreased side effects [151]. Benefiting from the homing property to parent tumor cells, loading paclitaxel into the exosomes from PANC-1 cell line with surface modifications could achieve better efficiency than free paclitaxel [152]. Another recent study suggested that loading paclitaxel and gemcitabine monophosphate into the engineered exosomes might have superior anticancer effects than loading gemcitabine alone [153]. Furthermore, Zhao et al. established a novel drug delivery system based on M1 macrophage-derived exosome, which provided an effective therapeutic strategy against drug-resistant pancreatic cancer [154]. Additionally, exosomes derived from bone marrow mesenchymal stem cells (BM-MSCs) have natural anticancer properties via circ_0030167 [141]. BM-MSCs exosomes could also be loaded with miR-124, which targeted EZH2 in PCCs and sensitized pancreatic cancer to chemotherapy in vitro and in mice models [155]. Recently, Hasoglu et al. firstly reported that pancreatic islet-derived exosomes can selectively kill pancreatic cancer cells without harming healthy cells [156]. These advancements underscore the promising potential of exosome-based therapies in enhancing the efficacy and specificity of pancreatic cancer treatments.

Engineered exosomes were promising in immunotherapy as well [2]. Galectin-9 expressed in PCCs promoted M2 phenotype of macrophages and immunosuppression. In the meanwhile, some chemotherapeutic drugs such as oxaliplatin could induce immunogenic PCCs death and activate antitumor immune response. After loading oxaliplatin and antisense oligonucleotides targeting Galectin-9 into exosomes which were originated from MSCs, they exhibited superior antitumor effects than oxaliplatin and Galectin-9 inhibition alone in mice models with orthotopic tumor [157]. Another recent study showed that loading immunogenic peptides and CCL22 siRNA into exosomes could expand CD8 + T cells and repress regulatory T cells within tumor microenvironment upon intramuscular administration [158]. In addition, precise photodynamic treatment could also be realized via exosomes. Under photoacoustic imaging guidance, engineered exosomes with chlorin e6 (photosensitizer) generated high amounts of ROS within the tumor. Consequently, disrupted exosomes membrane components could stimulate the immune system as tumor antigens [159]. Given the key role of exosomes in constructing premetastatic niches, loading pirfenidone into PCC-derived exosomes could alleviate liver fibrosis and suppress liver metastasis [160].

Besides chemotherapy and immunotherapy, targeted therapy against oncogenic KRAS, the most important driver mutation in pancreatic cancer, has attracting amounts attention from oncologists and pharmacologists for over three decades. Despite the breakthrough of KRAS G12C inhibitors in treating non-small-cell lung cancer, other KRAS-targeted therapy still has poor clinical efficacy [161, 162]. Specifically delivering antisense oligonucleotides or CRISPR/Cas9 via exosomes to target mutant KRAS in PCCs may also be a novel way to treat pancreatic cancer [163]. In orthotopic mice models, exosomes derived from MSCs carrying short interfering RNA effectively suppressed tumor growth through targeting KRAS G12D, the most common mutation subtype in pancreatic cancer [112, 164]. Moreover, a relevant phase I clinical trial is currently activating (NCT03608631). Additionally, pancreatic cancer progression could also be restrained by other types of engineered exosomes, such as PCCs-derived Exosomes loading with short interfering RNA against P21-activated kinase 4 (PAK4) [165], and BM-MSCs loading with miR-1231 [166]. Exosome-based treatments for pancreatic cancer were summarised in Table 2.

**Table 2:** Overview of exosome-based treatments for pancreatic cancer

Therapeutic Approach	Exosome Source	Loading Agent	Key Findings	References
Chemotherapy				
Enhanced drug delivery	PCCs-derived exosomes	Gemcitabine	Superior tumor suppression, reduced side effects compared to free gemcitabine	[151]
Enhanced drug delivery	PANC-1 cell line exosomes	Paclitaxel	Better efficiency than free paclitaxel	[152]
Combined drug delivery	Engineered exosomes	Paclitaxel and gemcitabine monophosphate	Superior anticancer effects than gemcitabine alone	[153]
Drug-resistant cancer therapy	M1 macrophage-derived exosomes	Not specified	Effective against drug-resistant pancreatic cancer	[154]
Anticancer	BM-MSCs-derived exosomes	miR-124	Sensitized pancreatic cancer to chemotherapy	[155]
Anticancer	Islet-derived exosomes	Not specified	Superior anticancer effects without damaging healthy cells	[156]
Immunotherapy				
Enhanced immunotherapy	MSCs-derived exosomes	Oxaliplatin and antisense oligonucleotides	Superior antitumor effects than oxaliplatin and Galectin-9 inhibition alone	[157]
Immune system modulation	Engineered exosomes	Immunogenic peptides and CCL22 siRNA	Expanded CD8 + T cells, repressed regulatory T cells in the tumor microenvironment	[158]
Photodynamic treatment	Engineered exosomes	Chlorin e6	High ROS generation within tumor, stimulated immune system as tumor antigens	[159]
Targeted Therapy				
Oncogenic KRAS targeting	MSCs-derived exosomes	Short interfering RNA	Suppressed tumor growth through targeting KRAS G12D	[112]
Oncogenic KRAS targeting	PCCs-derived exosomes	Antisense oligonucleotides, CRISPR/Cas9	Potential novel treatment approach for pancreatic cancer	[163]
Oncogenic KRAS targeting	MSCs-derived exosomes	Short interfering RNA	Phase I clinical trial activating	NCT03608631
Other oncogene targeting	PCCs-derived exosomes	Short interfering RNA against PAK4	Suppressed pancreatic cancer progression	[165]
Other oncogene targeting	BM-MSCs-derived exosomes	miR-1231	Suppressed pancreatic cancer progression	[166]

However, there are many challenges of exosome-based therapy at present. Besides therapeutic RNAs and proteins, engineered exosomes also carried other molecules. Therefore, prion particles, oncogenes, and viral miRNAs might be co-delivered to recipient cells, producing side effects. Additionally, the normal intercellular communication mediated by endogenous exosomes might also be interfered by exogenous exosomes [146]. In general, exosomes-based therapy is promising but remains further study.

### Exosome characterization techniques

Extracellular vehicles, including exosomes, can be characterized using a variety of advanced techniques [167]. High-resolution imaging methods like scanning electron microscopy (SEM), transmission electron microscopy (TEM), cryo-electron microscopy (Cryo-EM), and atomic force microscopy (AFM) are used for morphological characterization to visualize EVs’ structural features [168–170]. Proteomic analysis through mass spectrometry, Western blot, and enzyme-linked immunosorbent assay (ELISA) identifies key proteins such as CD9, CD63, and CD81 [171], ensuring purity and origin assessment. Particle size analysis of exosomes is mainly performed by dynamic light scattering (DLS), nanoparticle tracking analysis (NTA), and flow cytometry, these techniques measure the size distribution and concentration of exosomes, thus further characterizing the physical properties of exosomes [172, 173]. The lipidomics characterization of exosomes is conducted using chromatography techniques combined with mass spectrometry (LC-MS/MS). This approach enables the analysis of the lipid composition of exosomes, revealing their role in the structure and function of cell membranes [174, 175]. Emerging techniques, such as single-exosome detection methods, are also being developed to enhance the precision and detail of exosome analysis [174, 176]. Notably, innovative isolation techniques such as microfluidic technology, immunoaffinity capture, and resistive pulse sensing have been highlighted [177, 178]. These methods offer higher purity and specificity in isolating extracellular vesicles, thereby improving the reliability of subsequent analyses.

When applying these characterization techniques to different tissue types, the extraction methods may need to be adjusted based on the tissue’s specific properties. For example, the dense nature of pancreatic cancer tissue requires modifications to the standard protocols to effectively isolate exosomes. It is recommended to moderately increase the concentration of collagenase, the shaking speed, and the incubation time when extracting exosomes from pancreatic cancer tissue compared to other reported tissues such as gliomas and melanomas [76, 179, 180].

## Discussion

Tumor microenvironment is a highly heterozygous organization. Multiple cells and ECM coordinate with each other, synergistically promoting pancreatic cancer progression. Exosomes are vital mediators to realize rapid and abundant communication among different PCCs, CAFs, and immune cells within tumor microenvironment. Recently, a novel method showed that pure exosomes could be effectively isolated from tissues directly [181, 182]. Therefore, the role of exosomes in the tumor microenvironment are ready to be clear in the future study. Besides that, liquid biopsy via circulating exosomes is significant in diagnosis and stratifying patients to achieve individualized treatment. However, there are still some barriers restraining its clinical application. For example, exosomes could be extracted from blood or other body fluid through a variety of methods, such as ultracentrifugation and size exclusion chromatography, which may contribute to unrepeatable results in different clinical studies. Therefore, clinical standard in isolating exosomes from body fluid remains to be worked out.

The development of standardized protocols and methodologies is paramount to advancing EV research in pancreatic cancer. Consistent and reproducible results across different studies are essential to validate the potential of EVs as reliable biomarkers and therapeutic agents [144, 183]. Current research suffers from variability in isolation techniques, characterization methods, and analytical approaches [183]. Establishing consensus guidelines for these processes will enhance the comparability of data and facilitate the translation of EV-based diagnostics and therapies into clinical practice.

To validate the diagnostic potential of EV liquid biopsies, multicenter studies with large sample sizes are crucial [184, 185]. Such studies can provide robust evidence of the sensitivity and specificity of EV-derived biomarkers in detecting pancreatic cancer at various stages. There is also an urgent need to expedite Phase I and Phase II clinical trials to assess the safety, efficacy, and optimal delivery methods of engineered exosome-based therapy [186, 187]. Early-phase clinical trials will provide critical insights into the therapeutic potential of exosome and identify any potential challenges that need to be addressed before large-scale implementation [188].

In addition to exosomes, circulating DNA (cfDNA), metabolites, and circulating RNA (cfRNA) offer complementary insights into the tumor microenvironment and tumor burden [189, 190]. Combining these biomarkers in a multi-omics approach can enhance the sensitivity and specificity of liquid biopsies. Utilizing integrated multi-omics data models can provide a comprehensive view of the molecular landscape of pancreatic cancer, improving diagnostic accuracy and enabling precise patient stratification for personalized treatment [191].

## Conclusion

Exosomes could carry a variety of cargos including proteins, RNAs, metabolites, mediating the communication among heterogeneous cellular components within tumor microenvironment or even between different organs. The true potential utility of extracellular vesicles, particularly exosomes, lies in their ability to serve as both diagnostic biomarkers and therapeutic agents. Engineered exosomes are promising to implement targeted therapy, and circulating exosomes in plasma could be detected as liquid biopsy to accurately diagnose or monitor pancreatic cancer patients. However, for this promise to become a reality, additional work is needed. Standardization of isolation and characterization techniques, large-scale multicenter validation, and early-phase clinical trials are essential steps. Combining exosomal biomarkers with other biological materials like circulating DNA (cfDNA), metabolites, and circulating RNA (cfRNA) in a multi-omics approach can further enhance their clinical utility. These efforts will pave the way for innovative biomarkers discovery and the development of effective therapeutic strategies, ultimately improving patient outcomes in pancreatic cancer.

## Data Availability

No datasets were generated or analysed during the current study.

## Abbreviations

PDAC: Pancreatic Ductal Adenocarcinoma
TME: Tumor Microenvironment
EVs: Extracellular Vesicles
MVB: Multivesicular Body
PCC: Pancreatic Cancer Cells
MDSC: Myeloid-Derived Suppressor Cells
DC: Dendritic Cells
NK: Natural Killer cells
miRNA: MicroRNA
HSP: Heat Shock Protein
GPC1: Glypican 1
EpCAM: Epithelial Cell Adhesion Molecule
KRAS: Kirsten Rat Sarcoma Viral Oncogene Homolog
cfDNA: Cell-Free DNA
cfRNA: Cell-Free RNA
GLUT4: Glucose Transporter Type 4
TLR4: Toll-Like Receptor 4
CREB: cAMP Response Element-Binding Protein
IL-8: Interleukin 8
VEGF-C: Vascular Endothelial Growth Factor C
TNF-α: Tumor Necrosis Factor Alpha
IL-12: Interleukin 12
EMT: Epithelial-Mesenchymal Transition
CD: Cluster of Differentiation
LncRNA: Long Non-Coding RNA
RNA: Ribonucleic Acid
CD63: Cluster of Differentiation 63
CD81: Cluster of Differentiation 81
FGFRL1: Fibroblast Growth Factor Receptor Like 1
PI3K: Phosphoinositide 3-Kinase
AKT: Protein Kinase B
mTOR: Mechanistic Target of Rapamycin
XIAP: X-Linked Inhibitor of Apoptosis Protein
circRNA: Circular RNA
ROBO1: Roundabout Guidance Receptor 1
SRGAP2: SLIT-ROBO Rho GTPase Activating Protein 2
LN: Lymph Node
cfMiRNA: Cell-Free MicroRNA
SNORA: Small Nucleolar RNA
ADAM8: A Disintegrin and Metalloprotease Domain 8
ZIP4: Zinc Transporter 4
PCSK1/3: Proprotein Convertase Subtilisin/Kexin Type 1/3
RNF20: Ring Finger Protein 20
STAT3: Signal Transducer and Activator of Transcription 3
FOXO1: Forkhead Box O1
CRISPR/Cas9: Clustered Regularly Interspaced Short Palindromic Repeats/CRISPR Associated Protein 9
MSC: Mesenchymal Stem Cells
PAK4: p21-Activated Kinase 4
BM-MSCs: Bone Marrow Mesenchymal Stem Cells
NTA: Nanoparticle Tracking Analysis
DLS: Dynamic Light Scattering
SEM: Scanning Electron Microscopy
TEM: Transmission Electron Microscopy
Cryo-EM: Cryo-Electron Microscopy
AFM: Atomic Force Microscopy
LC-MS/MS: Liquid Chromatography–Mass Spectrometry/Mass Spectrometry
PBMC: Peripheral Blood Mononuclear Cells
FOLFIRINOX: A combination chemotherapy regimen including Folinic Acid (Leucovorin), Fluorouracil (5-FU), Irinotecan, and Oxaliplatin
GIP: Glucose-Dependent Insulinotropic Peptide
GLP-1: Glucagon-Like Peptide-1
ADCY1: Adenylate Cyclase 1
EPAC2: Exchange Protein Directly Activated by cAMP 2
ER: Endoplasmic Reticulum
ECM: Extracellular Matrix
